# Breast masses in childhood: a single-center experience

**DOI:** 10.3389/fped.2026.1759715

**Published:** 2026-02-03

**Authors:** Aytül Temuroğlu, Gökalp Rüstem Aksoy, Mine Özşen, Arif Nuri Gürpınar, Betül Berrin Sevinir

**Affiliations:** 1 Department of Pediatric Oncology, Bursa Uludag University, Bursa, Türkiye; 2 Department of Pathology, Bursa Uludag University, Bursa, Türkiye; 3 Department of Pediatric Surgery, Bursa Uludag University, Bursa, Türkiye

**Keywords:** breast masses, breast surgery, fibroadenoma, pediatric, phyllodes tumor

## Abstract

**Objective:**

Pediatric breast masses are rare conditions. Although most of them are benign, they can cause concern in families. The present study aims to determine breast masses’ clinical and pathological outcomes in childhood.

**Material and method:**

The records of patients who underwent further evaluations for breast masses between 2010 and 2023 at a single center were retrospectively reviewed.

**Results:**

A total of 32 patients with breast tumors were included in the study. The median age of the patients was 16 years (1-18 years); 90.6% (*n* = 29) were female, and 9.4% (*n* = 3) were male. Most patients, 90.6% (*n* = 29), had a painless, palpable mass. A family history of breast cancer was present in four patients. One patient had received chemotherapy for neuroblastoma and one for teratoma. The most common location was the upper outer quadrant in 35.5% of patients (*n* = 11). Bilateral mass involvement was present in five patients (15.6%). The mean tumor size was 32.64 ± 17.4 mm (range 9–80 mm). The mean tumor diameter was 24.6 ± 14.2 mm in patients who were followed without surgery and 39.2 ± 17.4 mm in those who underwent surgery (*P* = 0.017). A biopsy was performed in 53.1% (*n* = 17) of the patients, and surgery in 56.2% (*n* = 18). The most frequent pathology was fibroepithelial lesion and fibroadenomas 57.1% (*n* = 20). Malignant tumors (leiomyosarcoma and T cell lymphoma) were observed in 6.3% of the patients (*n* = 2) and borderline phyllodes tumors in 18.7% (*n* = 6). Recurrence was observed in 18.7% of the patients (*n* = 6) during the follow-up.

**Conclusion:**

In childhood, the most commonly encountered breast tumors are benign. However, careful monitoring is crucial due to the potential occurrence of malignant tumors. Further evaluations should be undertaken in patients with a history of malignancy or radiotherapy, masses larger than 5 cm, or masses with progressive growth.

## 1 Introduction

Breast tumors in childhood are uncommon, accounting for less than 1% of all pediatric malignancies. The incidence of malignant tumors is even lower, with an estimated rate of 0.08 per 100,000 individuals under the age of 19 (1). Fibroadenomas represent the most prevalent benign breast tumors in children (2), while malignant tumors typically arise from the metastatic involvement of leukemia or lymphoma. The most common presenting symptom is a painless, palpable mass. In adult patients, a family history of malignancy is considered a risk factor for the development of breast cancer. However, the significance of this condition has not been demonstrated for patients in childhood. Despite the majority of patients being benign, concern among families regarding this matter prompts further evaluations.

In the diagnosis of pediatric breast tumors, the preferred imaging modality is ultrasonography. Mammography is not utilized due to the radiation effect and the inability to obtain quality images in children. The American College of Radiology has recommended the Breast Imaging Reporting and Data System (BI-RADS) to standardize imaging terminology (3). However, while widely used in adults, this system has not demonstrated sufficient efficacy in children (4, 5). Magnetic resonance imaging is reserved for suspicious patients where ultrasonography findings are inconclusive.

The treatment of pediatric breast tumors remains controversial. Surgical intervention is recommended in patients with rapid growth or persistent masses, a history of radiotherapy or malignancy, or tumors exceeding 5 cm in size (6). In the current study, we aimed to investigate the clinical characteristics of patients presenting to our clinic with complaints of breast masses.

## 2 Materials and methods

### 2.1 Patients

This retrospective study included pediatric patients aged 0–18 years who presented with a palpable breast mass or swelling to the Pediatric Oncology Clinic of Uludağ University Faculty of Medicine between January 1, 2010, and December 31, 2023. Consecutive cases were identified from the institutional medical database.

### 2.2 Ethical aspects

The study protocol was approved by the Uludağ University Faculty of Medicine Ethics Committee on 01.07.2024 (Approval No: 2024-7/13). All procedures were conducted in accordance with the Declaration of Helsinki. As this was a retrospective study, informed consent was waived by the committee.

### 2.3 Study outcomes

The primary outcome was to determine the clinical, radiological, and pathological characteristics of pediatric breast masses. Secondary outcomes included assessing recurrence and histopathological distribution patterns.

### 2.4 Study design and definitions

This was a retrospective, descriptive study. Inclusion criteria were:

Patients aged 0–18 years,
Presentation with a breast mass confirmed by clinical examination or imaging, and
Availability of complete clinical and imaging records.

Exclusion criteria were:

Prior breast surgery or trauma,
Systemic disease that could affect breast tissue, and
Incomplete medical data.

Data completeness was verified by cross-checking electronic medical records, radiology archives, and pathology reports.

### 2.5 Description of procedures

For all patients, demographic characteristics, presenting symptoms, family history, lesion laterality and location, imaging findings (ultrasonography, MRI), BI-RADS classification, biopsy and/or surgical procedures, and pathology results were recorded. Imaging reports were reviewed by pediatric radiologists. BI-RADS categories were assigned according to the latest ACR guidelines.

### 2.6 Follow-up

Follow-up data, including the date of last evaluation and presence of recurrence, were obtained from outpatient clinic records. Patients were routinely monitored every 6–12 months after diagnosis or surgery, depending on clinical need.

### 2.7 Statistical analysis

All statistical analyses were performed using SPSS version 28.0 (IBM Corp., Armonk, NY, USA). Continuous variables were tested for normality using the Shapiro–Wilk test and presented as mean ± standard deviation (SD) or median (minimum–maximum). Categorical variables were compared using the chi-square or Fisher's exact test. A *p*-value < 0.05 was considered statistically significant.

## 3 Results

Thirty-two patients who presented to the pediatric oncology outpatient clinic with a breast mass for 13 years were included in the study. The median age of the patients was 16 years (range: 1–18 years), with 90.6% (*n* = 29) being female and 9.4% (*n* = 3) being male. Twenty eight (87.5%) patients were over 10 years old. At admission, 90.6% (*n* = 29) of the patients presented with painless masses. Additionally, two patients had complaints of nipple discharge along with the palpable mass, and three patients presented with only breast enlargement.

Four out of the 32 patients had a family history of breast cancer. One patient was under surveillance due to Cowden syndrome with PTEN mutation. A chemotherapy history was present in 6.2% (*n* = 2) of the patients (due to neuroblastoma and immature teratoma).

The most common site of localization was the upper outer quadrant, observed in 34.4% (*n* = 11) of the patients. Bilateral masses were found in 15.6% (*n* = 5) of the patients. The demographic characteristics of the patients are presented in Table 1.

**Table 1:** Demographic characteristics of the patients.

Characteristic	n (%)
Female	29 (90.6)
Male	3 (9.4)
Mean age at diagnosis (year)	16 (1–18)
Family history of breast cancer	4 (12,5)
History of malignancy	2 (6,3)
Mean tumor size (mm)	32.64 ± 17.4
Positive genetic abnormality	1 (3,1)
Tumor localization	
Upper outer quadrant	11 (34.4)
Retro areolar region	7 (21.9)
Upper inner quadrant	5 (15.6)
Multiple quadrants	5 (15.6)
Other areas	4 (12.5)

All patients underwent ultrasonography, with magnetic resonance imaging additionally performed in seven patients. According to the BI-RADS classification system, evaluation was available for six patients, with four patients classified as category 3 and two patients as category 4. The mean tumor size was 32.64 ± 17.4 mm (range: 9-80 mm).

**Figure 1:**
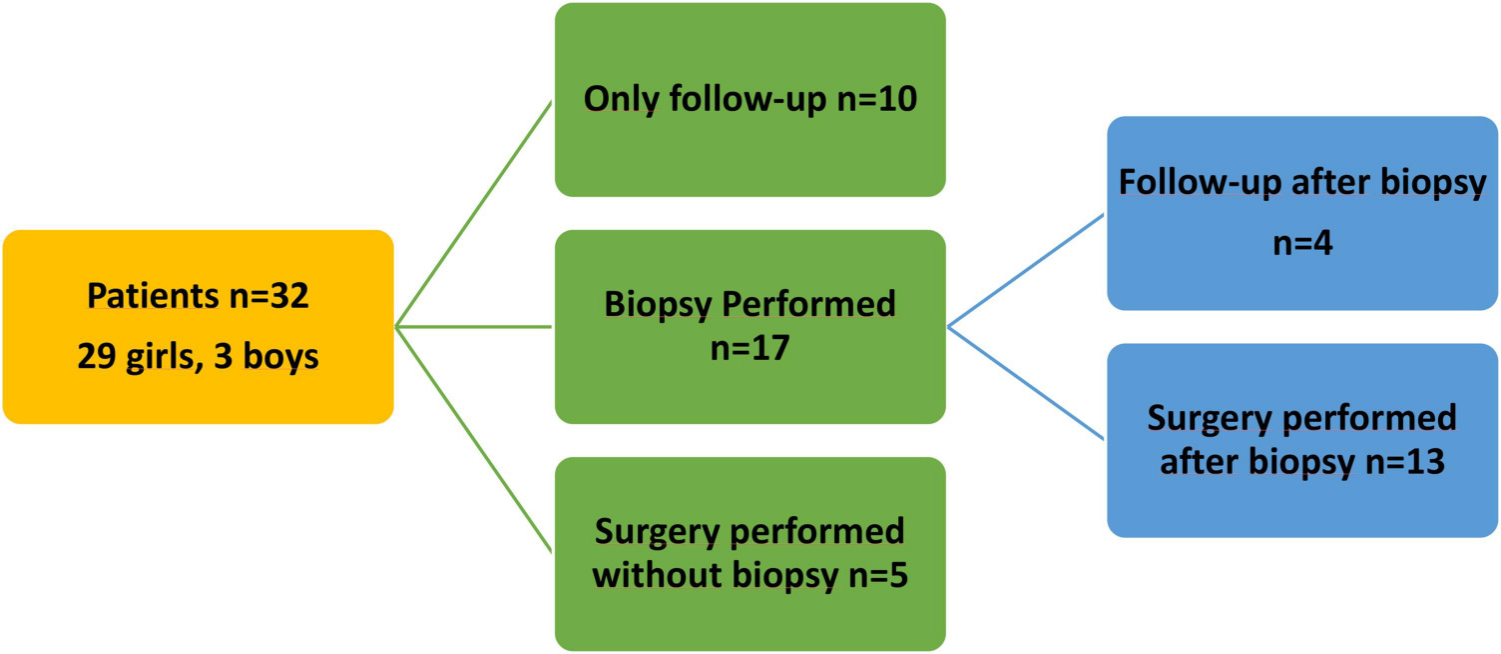
Treatment algorithm used for the patients.

A biopsy was performed on %53 (*n* = 17) patients (core needle biopsy in nine and fine needle aspiration in eight), and surgical intervention was performed on 18 (58%) patients. Thirteen of the patients who underwent a biopsy were subsequently taken to surgery. Surgery was performed without a biopsy in five patients. The reasons for surgery in 5 patients who underwent surgery without a biopsy were that the masses were very large and appeared solid at the time of admission. Among the patients undergoing surgery, 77.8% (*n* = 14) underwent mass excision, 16.7% (*n* = 3) underwent mass excision and partial mastectomy, and 5.5% (*n* = 1) underwent lumpectomy and mass excision. Local excision or lumpectomy was preferred for well-circumscribed benign or borderline lesions, while mastectomy was reserved for selected cases with extensive disease, unfavorable breast-to-tumor size ratio, or strong suspicion of malignancy. Our approach to the patients is illustrated in Figure 1.

Surgery was planned for 16 patients due to the large tumor size at diagnosis or rapid growth during follow-up. Two patients underwent surgery due to a family history of malignancy. The mean tumor diameter was 24.6 ± 14.2 mm in patients who were followed up without surgery and 39.2 ± 17.4 mm in those who underwent surgery. There was a statistically significant difference in tumor sizes between patients who underwent surgery and those who did not (*p* = 0.017). A moderate positive correlation was found between lesion size and the likelihood of undergoing surgery (r = 0.445, *p* = 0.011), indicating that larger lesions were significantly more likely to be treated surgically (95% CI: 0.114–0.687).

**Figure 2:**
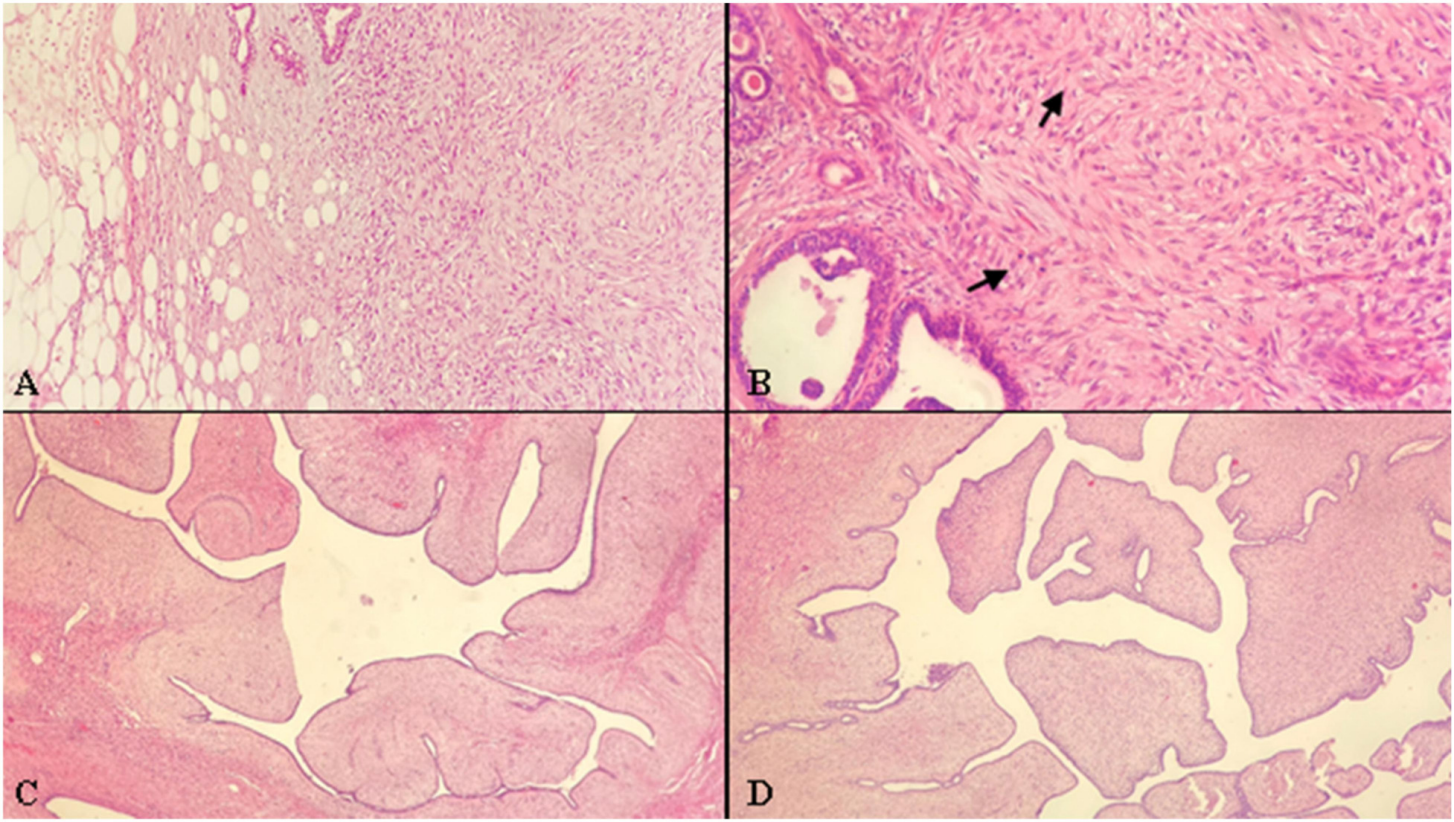
**(A)** infiltrative tumor margins in borderline filloid tumor (H&E x200), **(B)** presence of mitotic activity in the stroma of borderline filloid tumor (indicated by arrow, H&E x400), **(C,D)**. Leaf-like growth pattern in benign filloid tumor (H&E x200).

The most common pathology result among the patients undergoing biopsy was reported as fibroepithelial lesions (*n* = 10, 58.8%). Among those undergoing surgery, the most common pathology result was phyllodes tumors (*n* = 8, 44.4%) (borderline in six, benign in two) (Figure 2).

Following this, fibroadenomas were observed in (*n* = 7, 38.8%) of patients (juvenile fibroadenoma in four, fibroadenoma in two, and myxoid fibroadenoma in one). The pathology results are presented in Table 2. Patients with BI-RADS 4 category received diagnoses of juvenile fibroadenoma and leiomyosarcoma.

**Table 2:** Pathology (biopsy and surgery) results of the patients.

Pathology results(biopsy and surgery)	*n* = 35 lesions from 32 patients (%)
Fibroepithelial lesion	10 (28.5)
Fibroadenoma	5 (14.2)
Juvenile fibroadenoma	4 (11.4)
Myxoid fibroadenoma	1 (2.9)
Phyllodes tumors:	
Borderline	6 (17)
Benign	2 (5.7)
Others	7 (20)

Others: Leiomyosarcoma, Non-Hodgkin lymphoma, Adenosis, Papillary lesion, Pseudoangiomatous stromal hyperplasia, Ductal ectasia, Gynecomastia.

The mean tumor size was 44.8 ± 13.9 mm for fibroadenomas and 41.4 ± 19.3 mm for phyllodes tumors. The maximum tumor size, 8 cm, was observed in a patient with a phyllodes tumor. When compared in terms of tumor diameter, there was no significant difference in the size of masses between the most commonly seen phyllodes tumors and fibroadenomas (*p* > 0.05). Of the two patients who did not undergo biopsy, one had a hemangioma, and the other had a lymphangioma.

The median follow-up duration was 14 months (1-124 months). Recurrence was observed 18.7% (*n* = 6) in three patients with juvenile fibroadenoma, one patient with myxoid fibroadenoma, and two patients with borderline phyllodes tumors during the follow-up. The short follow-up period of the patients was due to the fact that patients with benign tumors stopped following up after a while. After recurrence, a biopsy was performed on two patients, and the result was reported as a fibroepithelial lesion. Surgical intervention was not considered.

## 4 Discussion

Adults with a family history of malignancy are at significantly increased risk of developing breast cancer. However, there is insufficient data to support such assertions in children. Although the likelihood of malignant tumors occurring in adolescents is very low, individuals with a family history tend to undergo biopsy and surgery at higher rates (7). Four of our patients had a family history of ductal carcinoma. Two patients were managed with follow-up alone, while the other two underwent surgery. Fibroadenoma was detected in one patient, and a benign phyllodes tumor was found in the other. Radiotherapy or a history of previous malignancy increases the risk of breast cancer (8). Two patients had a history of primary malignancy. Pathologic evaluation of these patients resulted as papillary lesion and juvenile fibroadenoma.

The approach algorithms for childhood breast tumors have not been clearly delineated. They are generally shaped by the clinician's experience, radiological data, and the patient's condition. The BI-RADS system, which is frequently utilized for diagnosis in adult patients, has not been commonly adopted in children. In a cohort study conducted by Lawrence et al. with 453 patients, it was observed that the BI-RADS scoring system may not be suitable for children and that this system is mainly used in patients evaluated in non-pediatric facilities (9). In another study conducted on children, 283 patients were included, and it was observed that all patients with BI-RADS 4 had benign lesions (5). BI-RADS results were available for 18.7%(*n* = 6) of our patients. Among the patients with a 4 category, one received a diagnosis of a malignant tumor. This suggests there may be a need for the development of systems specific to children.

While benign breast tumors are commonly observed in children, their sizes can significantly increase, leading to concerns. Surgical intervention is recommended when the tumor size exceeds 5 cm or does not diminish over a three- to six-month follow-up period (10, 11). Our patients undergoing surgery exhibited statistically significantly more significant tumor sizes compared to those managed conservatively. Surgical decisions were made for male patients due to the lack of reduction in tumor size during follow-up and for two patients with a family history of malignancy. In the cohort study conducted by Westfal et al., it was reported that unnecessary intervention was performed in 81.1% of 1909 patients, and the follow-up period of the patients could be increased up to 90 days (12). Since the algorithms for approaching patients presenting with breast masses in adolescence are not sufficient, adult algorithms can be used. The anxious approach of the families may affect the surgical decision. We posit that the collaborative management of pediatric breast tumor patients by a multidisciplinary team consisting of pediatric surgeons, oncologists, psychiatrists, and general pediatric specialists may mitigate surgical indications. Although surgery is the mainstay of treatment, radiotherapy and chemotherapy are used in malignant tumors (13).

**Figure 3:**
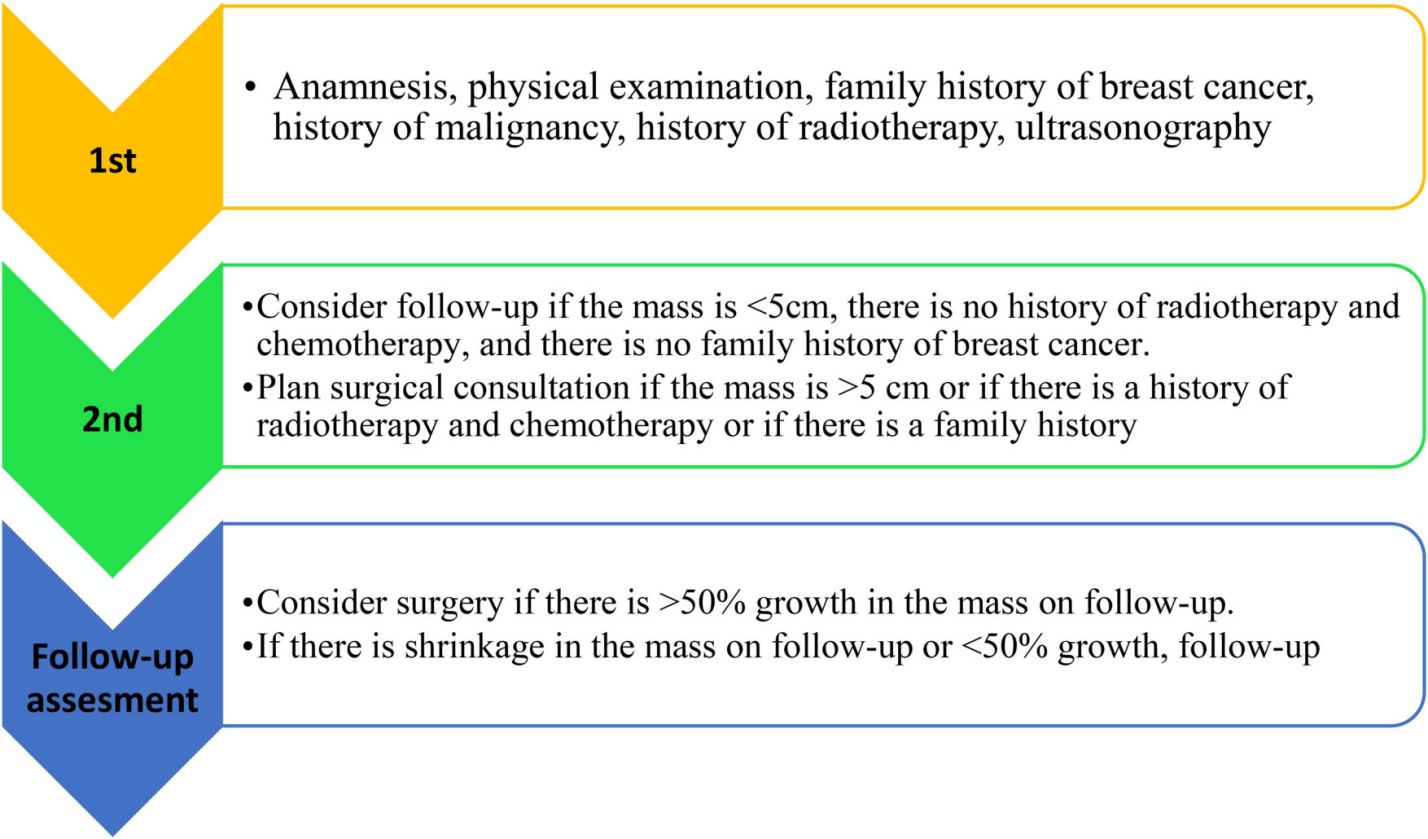
Approach to the patient presenting with pediatric breast mass **(**15).

Pediatric breast cancers are diagnosed with more advanced stages and larger masses compared to adults (14). In our series, chemotherapy was given only to the patients diagnosed with non-Hodgkin lymphoma. No patient received radiotherapy. A pathway that can be used to approach a patient presenting with a pediatric breast mass is shown in Figure 3. This algorithm requires validation in larger, multi-center cohorts. According to this algorithm, the indications for our patients who underwent surgery were tumor size, rapid growth of the tumor, and a family history of malignancy.

Ultrasonography is the most commonly used method in the differential diagnosis of childhood breast tumors. Ultrasonography can distinguish between cysts and solids, identify vascularization, and determine mass morphology. Other advantages include its easy accessibility and lack of radiation exposure. However, it has been observed that a large mass size does not increase the likelihood of the mass being malignant (16). For this reason, the algorithm shown in Figure 3 can be considered a proposal made according to the literature. While a 20% growth rate during follow-up is considered significant in adults, this rate was set at 50% in children (15, 17). This may be due to changing hormone levels and the higher prevalence of benign pathologies. Therefore, we believed it would be more appropriate to add a 50% growth threshold to the algorithm.

In childhood, fibroadenomas are the most frequently encountered breast tumors, followed by phyllodes tumors. Fibroadenomas constitute approximately 50% of breast tumors, and some may regress spontaneously. They typically present as slow-growing multiple masses. Juvenile fibroadenomas, on the other hand, are rapidly growing fibroadenomas observed in adolescents. They grow faster than other tumors and can reach sizes of 5-10 cm. Distinguishing these tumors from phyllodes tumors using imaging techniques or a core needle biopsy is challenging (18). Among our patients diagnosed with fibroadenomas, 42.8% (*n* = 3) had fibroadenomas, 42.8% (*n* = 3) had juvenile fibroadenomas, and 14.4% (*n* = 1) had a myxoid fibroadenoma. Two patients with recurrent juvenile fibroadenomas had significantly large tumor sizes and tumors at the surgical margin. Recurrence was observed in the contralateral breast of patients with myxoid fibroadenomas and juvenile fibroadenomas, and these patients were managed with biopsy and follow-up.

The World Health Organization classifies phyllodes tumors as benign, borderline, and malignant (19). They grow rapidly and can reach larger sizes than fibroadenomas. They are the second most common malignant breast tumors after invasive carcinomas (20). Recurrence can occur in various proportions across all histological types of phyllodes tumors (21). The absence of a tumor at the surgical margin is important for preventing recurrence (22). Wide resection is not recommended in benign phyllodes tumors due to the low risk of recurrence (23). Borderline and malignant tumors should be operated with a wide surgical margin (24). Of our patients, 22.7% (*n* = 7) received a diagnosis of phyllodes tumors. Recurrence was observed in the contralateral breast in one patient with a borderline phyllodes tumor, as well as in the same breast in another patient with a phyllodes tumor. The surgical margin status of our patients' Phyllodes tumors was tumor negative.

Leiomyosarcoma is a rare tumor among breast tumors, with reported patients primarily documented in the literature as adult presentations (25). One of our patients involved leiomyosarcoma. No evidence suggestive of metastasis was found in the screenings of the patient who underwent total surgical excision; thus, close follow-up was initiated. Our patient represents the first presentation of leiomyosarcoma in childhood reported in the literature.

Pseudoangiomatous stromal hyperplasia is characterized by myofibroblastic proliferation observed in the breast. It can give rise to benign tumors (24). During differential diagnosis, they cannot be distinguished from fibroadenomas by using imaging techniques. Surgical treatment is recommended if excessive growth occurs during follow-up one of our patients presented with bilateral breast enlargement and a mass in one breast. The patient was also undergoing treatment for hyperprolactinemia and epilepsy. The pathology results indicated pseudoangiomatous stromal hyperplasia. Surgical intervention was performed; however, postoperative recurrence of breast enlargement was observed. Three years after the initial operation, the patient required mastectomy two more times.

In male children, benign conditions are frequently observed. Among our male patients, one presented with ductal ectasia, while another had gynecomastia. In addition, one patient received a diagnosis of T-cell lymphoma. Lymphomas in childhood are exceedingly rare occurrences. Secondary lymphomas are more common than primary lymphomas (26). Breast involvement by B-cell lymphoma is most prevalent among non-Hodgkin lymphomas, whereas T-cell lymphoma is exceedingly rare. There are patient reports documenting such occurrences in the literature (27). T-cell lymphomas are more commonly observed in girls. The diagnosis of primary lymphoma entails the absence of lymphoma involvement in other areas, no prior diagnosis of lymphoma, and sufficient breast tissue for lymphoma diagnosis (28). In our study, the patient diagnosed with lymphoma was evaluated as having secondary breast lymphoma due to the presence of a mediastinal mass. Chemotherapy was initiated, and no recurrence was observed during the patient's follow-up.

Infantile hemangiomas are the most common benign vascular tumors in children. Propranolol is used as the first choice in the treatment of infantile hemangioma. Propranolol is a beta blocker that was first used in 2008 when it was noticed that hemangiomas were regressing in a patient who was treated for cardiomyopathy (29). Since many patients have the potential to heal spontaneously, they often do not require treatment. However, treatment may be necessary in approximately 10% of patients where complications develop. Hemangiomas localized to the breast may lead to breast hypoplasia; thus, it is recommended to initiate treatment (30–32). Propranolol was considered for hemangiomas but not initiated due to post-proliferative phase presentation. One patient was evaluated at the age of 11 during the last follow-up, showing Tanner stage 2-3 development without observed breast hypoplasia. We did not encounter any complications such as breast hypoplasia in the follow-up of our patients with breast hemangioma.

Breast cancer is exceptionally rare in children, with invasive breast carcinomas being the most commonly encountered malignant breast tumors (33). Of our patients, 6.3% (*n* = 2) were diagnosed with malignant breast tumors (leiomyosarcoma and lymphoma), while six patients received a diagnosis of borderline phyllodes tumors. None of our patients presented with invasive breast carcinoma. Although breast cancer is rare in children, it is known to cause anxiety in patients and their families.

Many studies have been conducted on the psychological effects of breast cancer or surgery in adult patients (34). These studies may also be needed in children. Pediatric patients may experience anxiety similar to adults, necessitating psychological support and family counseling. Psychological problems have been observed more frequently in individuals who have been treated for solid tumors and survived compared to their siblings (35). Although breast tumors are often benign, undergoing pediatric oncology follow-up, biopsy, or surgery can negatively affect adolescents psychologically. Damage to breast tissue through biopsy or surgery, the risk of malignant tumor formation, and the fact that the mass is growing even if it is benign can lead to this situation. Fine-needle aspiration (FNA) is no longer considered a standard diagnostic tool for breast lesions in adult patients. However, in pediatric populations, FNA may be selectively used in carefully chosen cases to minimize tissue trauma. Nevertheless, its limitations, including limited architectural assessment and reduced diagnostic accuracy compared to core needle biopsy, should be acknowledged. It may be important to treat these patients as sensitively as possible during examination, to state that the risk of malignancy is very low, and to carefully determine the control intervals. Data regarding the anxiety levels of our patients could not be obtained from the hospital notes.

The observation in our study that surgical intervention was primarily required for larger or rapidly growing lesions is consistent with previous recommendations for the management of pediatric breast masses. The recurrence of juvenile fibroadenomas and borderline phyllodes tumors supports the idea that these lesions may exhibit more unpredictable biological behavior. In addition to tumor size, we also analyzed age, sex, and variables related to physiological breast development to explore potential differences between the surgery and observation groups. However, none of these parameters reached statistical significance in our cohort. However, due to the limited sample size, these trends should be interpreted with caution and cannot be confirmed by formal statistical tests.

As a retrospective study, some inherent limitations, including possible selection bias, heterogeneity in referral patterns, and differences in follow-up duration, should be acknowledged. Additionally, small sample size, which may limit the generalizability of the findings. Furthermore, because the study was conducted at a tertiary pediatric oncology center, the patient population may not fully reflect the general pediatric population.

## 5 Conclusion

While childhood breast tumors are predominantly benign, a small but clinically significant proportion of patients may present with phyllodes tumors or other rare malignant lesions. In this cohort, the main factors associated with surgical intervention were larger tumor size, rapid intermittent growth, and worrisome imaging features, while in select cases, a family history of malignancy and prior chemotherapy or radiotherapy influenced the decision-making process.

Our findings demonstrate the continued importance of careful clinical and radiological follow-up, particularly for fibroepithelial lesions and rapidly growing masses, which exhibited higher recurrence rates in this series. While the retrospective design and limited sample size limit the generalizability of the results, the study provides practical information on the evaluation and management of pediatric breast masses and highlights the need for prospective, multicenter studies to better define risk patterns and long-term outcomes.

## Data Availability

The raw data supporting the conclusions of this article will be made available by the authors, without undue reservation.
